# A cross-case analysis of developing program sustainability and institutionalization in early stages of a multisite biomedical student diversity initiative

**DOI:** 10.1186/s12909-021-02663-2

**Published:** 2021-05-03

**Authors:** Krystle Palma Cobian, Hector V. Ramos

**Affiliations:** 1 Jonathan and Karin Fielding School of Public Health, University of California, Los Angeles, USA; 2 School of Education and Information Studies, University of California, Los Angeles, USA

**Keywords:** Institutional transformation, Institutionalization, Program sustainability, STEM innovation, STEM education, Biomedicine

## Abstract

**Background:**

Grant funding often drives innovative programming in efforts to enhance diversity in biomedical fields, yet strategies for sustainability of grant-funded biomedical intervention are not well understood. Additionally, as funding agencies shift toward supporting institutional change to biomedical training, less is known about the extent to which sustainability strategies can support long-term institutionalization of the original goals of the grant-funded initiative. The purpose of this study is twofold: to identify strategies used by grant-funded programs for promoting sustainability, and to examine the interrelations between the concepts of sustainability and institutionalization during early stages of grant-funded biomedical career training efforts.

**Methods:**

We employed a multiple case study design and cross-case analysis using interviews of program administrators and participants from 10 undergraduate institutions that received Building Infrastructure Leading to Diversity (BUILD) awards funded by the National Institutes of Health (NIH).

**Results:**

BUILD sites engaged in the following strategies to develop program sustainability: 1) scaling and adapting to expand programmatic impact, 2) identifying additional funding and cost-cutting measures, 3) developing and maintaining infrastructure and structural operations, 4) leveraging relationships and with intra-and inter-institutional partners, and 5) and addressing hiring, policies, and reward systems at the institution. Senior administrative support supported program sustainability and early institutionalization, although we also identified situations where participants felt that they were on track for sustainable changes without administrative support or institutional change. Of the strategies identified, those that involve organizational and infrastructural changes contribute to early stages of institutionalization.

**Conclusions:**

This study contributes to literature on organizational change by providing evidence of distinctions and interrelations between program sustainability efforts and institutionalization of change efforts in that some sustainability strategies can overlap with strategies to move toward institutionalization. The findings indicate the importance of program administrators developing early sustainability plans that also lead to institutionalization, as well as an opportunity for funding agencies to develop technical assistance on sustainability, organizational change, and institutionalization as a resource to support program administrators’ efforts toward making lasting, structural change on their campuses.

**Supplementary Information:**

The online version contains supplementary material available at 10.1186/s12909-021-02663-2.

## Introduction

Grant funding often drives innovation in higher education, allowing initial project planning, pilot testing, and implementation of science, technology, engineering, and mathematics (STEM) program interventions [1, 2]. Yet, student-centered innovations may be short-lived, and often have limited success and adoption because they do not become part of an institution’s culture [3]. While “institutionalization, program sustainability, and repeatability sound like issues addressed during the final phases of a multi-year, several million dollar project, in fact they stand as some of the factors that need to be considered first, even prior to the initial, planning phases of the project”( [4])pp. 336–337). As a result, it is becoming common practice for funding agencies to require applicants to submit program sustainability plans as part of funding proposals [5, 6] and evaluate institutional commitments to gain a better understanding of how grant recipients conceptualize and plan for sustainability [7].

Several national grant-funded initiatives have been developed to support diversity, advance equity, and broaden participation in biomedical disciplines. For example, the National Institutes of Health (NIH), National Science Foundation (NSF), Association of American Colleges and Universities (AAC&U), and private foundations such as the Howard Hughes Medical Institute (HHMI) and the Robert Wood Johnson Foundation have allocated funds to support institutions in implementing science, technology, engineering, and mathematics (STEM) education innovations and institutional transformation. Sustainability, or continuing a program, is critical to maintaining momentum created by the brief infusion of external funds [5, 8].

While plans for sustainability need to be made early, there are also distinctions between sustainability and institutionalization that are important to consider in order to achieve program goals once external funding ends. Sustainability involves maintaining desired outcomes of grant-funded innovations, which may rely on different sources of outside funding [9], whereas institutionalization is a process and outcome of iterative shifts between practices that embed innovations into permanent parts of the institution [10–13]. What is still unclear is how recipients of grant-funded interventions successfully work toward sustainability, particularly during the early stages of program implementation [6]. Finally, since institutionalization differs slightly from the concept of program sustainability, it is critical to examine how biomedical training grant recipients consider institutionalization as a goal.

The purpose of this study is to examine how educational institutions are actively building program sustainability (i.e. maintaining a program and/or desired program outcomes) and engaging in institutionalization efforts (i.e. structurally embedding innovations from a program into permanent parts of the institution) during the early stages of program implementation. We identify how some institutions simultaneously approach the more complex process of institutionalization, requiring broader changes throughout the institution, and also probe how participants think about institutionalization as an aspect of broadening and sustaining change at their institution. We analyzed case study data from 10 Building Infrastructure Leading to Diversity (BUILD) sites during years three and four of a potential 10-year STEM program implementation. Funded by the National Institutes of Health (NIH), each undergraduate institution developed approaches intended to determine the most effective ways to engage students from diverse backgrounds in biomedical research, with the long-term goal of preparing an increased number of students to become potential future contributors to NIH-funded research [14]. By instituting effective interventions and strategies to diversify biomedical research, BUILD institutions aimed to contribute to broader transformational impact for their campuses. Considering the large investment among federal and private agencies to provide grant funding to support STEM interventions, campuses with grant-funded initiatives and funding organizations have considerable interest in seeking strategies and lessons on how to extend sustainable, student-centered program innovations for success in entering biomedical education and the workforce.

### Context of the NIH BUILD initiative

The NIH has funded training programs to support the development of a diverse biomedical workforce for nearly 45 years, with most of the funding focused on students [15]. The NIH-funded BUILD initiative was unique in that in addition to a range of student-focused activities and funding, sites also provide related faculty development, increase institutional research capacity, and work towards an increased commitment to diversity in biomedical majors [14, 16]. An underlying assumption of this endeavor is that by including institutional-level changes, innovations would be sustained and institutionalized. For a second five-years of funding, applicants were required to include a sustainability plan for each proposed activity and a decreased reliance on grant funds by 25% each year [16]. Campuses may have been less focused on this level of expectation in the preliminary funding phase when these data were initially gathered, but it was known by grantees from the beginning that the funding mechanism allowed no more than 10 years for the initiative.

### Sustainability and institutionalization in higher education

The concept of program sustainability comes primarily from health literature. Moore and colleagues [9] systematically developed a comprehensive sustainability definition: “after a defined period of time, the program, clinical intervention, and/or implementation strategies continue to be delivered and/or individual behavior change is maintained; the program and individual behavior change may evolve or adapt while continuing to produce benefits for individuals/systems” ( [9])p. 5). Scheirer [5] examined studies from the health field regarding the extent to which sustainability might be possible and under what conditions, and found that 60% or more of program sites sustained at least some of their activities after program funding ended. Additionally, Scheirer found that more than 75% of the studies noted the importance of leaders who could advocate effectively for the program. Evaluation findings often identified staff and external stakeholders’ belief in program benefits as important to sustainability, and community supporters also played a key role in helping secure resources and mobilizing support. This research suggest that program sustainability is focused on maintaining, and where necessary, evolving or adapting efforts as needed to continue the outcomes of an innovation, and that advocacy from leadership is critical for sustaining innovations.

In the health literature, sustainability of a grant-funded program is often modeled as the final stage of a program’s evolution. However, one study found sustainability processes in organizations (i.e. routinization) during early stages of program implementation [17], suggesting that stage models of sustainability may be deceiving since sustainability efforts are concomitant with program implementation [18]. Additionally, differing views of sustainability may also lead to different strategies to achieve it. For example, Johnson and colleagues [7] examined how researchers conceptualized and planned for the sustainability of health interventions for all active and completed U.S. NIH R01 Grants and equivalents between 2004 and 2016. The researchers found that grant recipients had widely varying views on sustainability, and few employed sustainability frameworks and/or resources to support their efforts to continue programs. While the study used the comprehensive definition of sustainability developed by Moore and colleagues [9], it did not examine interrelations between sustainability strategies and institutionalization.

*Institutionalization* is defined as the processes that embed innovations into permanent parts of the organization [10–12]. From a sociological perspective, institutionalization is “both a process and an outcome, representing the manner of attaining a social order that reproduces itself, as well as the state of having realized this order”( [13])p. 38). Institutionalization within higher education is often thought of as “a process through which innovations or novel practices are made to be normal or prevailing practices within the organization”( [19])p. 38). When educational innovations are institutionalized, they shift from being regarded as a special project to becoming an expected and vital part of the institution [20]. Institutional facilities (e.g., labs, classrooms, and study spaces), reward systems (e.g., policies and incentives to support faculty development), and behavioral norms (e.g. actual values that guide teaching and mentoring behaviors toward diverse students) are important for institutionalization [21, 22] because they include structural and behavioral processes that aim to achieve embeddedness within the organization.

Scholars have examined institutionalization in higher education broadly [10], as well as specifically with respect to transdisciplinary scholarship [23], diversity efforts [24, 25], pedagogical practices and curriculum implementation [26, 27], and special programs and initiatives [28–33]. Considerable literature on institutionalization offers specific guidance on how to achieve the goal of embedding policies and practices into the structure of an institution. For example, the Comprehensive Action Plan for Service Learning (CAPSL) model developed by Bringle & Hatcher [28, 33] emphasizes using resources to develop infrastructure (e.g., creating office space, budgets, and personnel) in order to successfully implement service learning and formalize it as a permanent part of the institution.

Studies and frameworks focused on *the process of institutionalization* point to environments and contextual factors for change [34–36], processes of change [37, 38], and strategies for implementing change [39, 40]. Indeed, “organizational change and its institutionalization are inexorably linked. Change is difference: institutionalization is making that difference last” ( [41])p. 6). The Multi-Faceted Framework for Understanding Change provides a useful framework to understand how values and institutional culture are important within the context of institutional change [42] This framework argues that institutions are values-driven and the values are *actual, espoused or aspirational.* Espoused and aspirational values are a reflection of the value goals of a campus, not necessarily reflecting actual behavior. Aspirational values may not manifest across the institution, yet such values can provide valuable targets for campuses to invest resources and efforts to actualize their objectives and goals [42]. Values are embedded in the institutional culture [43], which is defined as the stable and enduring part of daily higher education organizational practices and beliefs. For example, perceptions of support for teaching had the strongest effect on engineering faculty members’ sensitivity to ethnically underrepresented engineering students’ needs, suggesting that administrators must have a visible commitment (i.e. behaviors guided by actual values) to teaching to successfully sustain curricular and pedagogical changes [26].

Institutionalization can encompass culture change, but also requires changes in infrastructure [33]. The shift in cultural norms and behaviors is one dimension of achieving institutionalization, but must also include physical structures, policies, and curricula meant to make a difference that lasts.

### STEM-specific sustainability and institutionalization efforts

Sustainability and/or institutionalization efforts have been addressed by many with respect to externally-funded efforts to improve STEM education. Bailey and colleagues [44] studied sustainability and institutionalization, finding that most program sites worked to create materials and programs (i.e. an output-oriented perspective), versus changing the environment that gave rise to the issue in the first place (i.e. a process-oriented perspective). The researchers suggest that a process-oriented perspective is more likely to lead to institutionalization compared to output-oriented changes.

Strategies that involved basic operational or environmental changes that emphasize STEM curriculum and professional development proved more sustainable than strategies that increase courses or faculty, although the latter contribute to significant long-term change (i.e. institutionalization) [44]. Regarding successful undergraduate educational reform, a study of eight campuses identified strategies such as: shifting toward collective responsibility for curricula and teaching; hiring educational experts within STEM departments; reorganizing institutional structures; employing institutional data to provide evidence to support student learning; creating institutional business models to promote sustainability; managing high-quality teaching and research; and leveraging relationships with a higher education association to advance educational reforms and institutional change [45]. Finally, STEM intervention programs that struggle to gain financial or other forms of support from senior-level administrators are viewed as less valued by important university stakeholders and rarely institutionalized [46].

Overall, the literature suggests that sustainability involves maintaining, and where necessary, evolving or adapting efforts to continue the outcomes of an innovation. Support leadership is critical for sustainability, and differing views and conceptions of sustainability from program implementers may pose a challenge in successfully sustaining a program. Institutionalization is a process and outcome of iterative shifts between policies, practices, and aspirational values aimed at embedding an innovation as a permanent part of the institution’s actual values and practices. The literature rarely takes into account the strategies that STEM program administrators employ in early stages of program implementation to begin the process of sustainability and/or institutionalization. Additionally, the literature is limited in its focus on teaching and curricular reforms, rather than a multi-pronged and comprehensive approach with extracurricular program implementation and goals. BUILD sites differ in that they were engaged in multiple simultaneous efforts focused on supporting underrepresented students and biomedical faculty through changes in curricula, faculty grant-writing training, mentorship training, student research opportunities, and developing partnerships with community colleges and research universities. Little is known about how program administrators think about and consider sustainability and institutionalization, as well as the strategies they use to begin the process of institutionalization and program sustainability as they implement early phases of building program interventions. To address this gap in understanding, the purpose of this study is to examine how educational institutions are actively building program sustainability and engaging in institutionalization efforts during the early stages of program implementation.

## Methods

### Analytic approach: multiple case study design

To understand how BUILD sites are working towards program sustainability, the research team employed a multiple case study approach [47, 48]. Case study research is a predominantly qualitative technique in which a bounded system (a “case”) or multiple bounded systems are studied at length in their real-life context(s) [48]. Case studies are useful for description of context-specific implementation of practices including explanation, exploration, and replication. A multiple case study design generates findings from cluster comparisons that represent the larger phenomenon, known as the “quintain” [47]. Multiple case studies allowed us to understand how different institutional contexts shaped BUILD program sustainability efforts, as well as how institutionalization strategies converge and diverge across cases.

### Case selection

The data are comprised of interviews with administrators, faculty, staff, and students at 10 Building Infrastructure Leading to Diversity (BUILD) sites. In FY 2014, 10 NIH BUILD awards were issued to undergraduate institutions across the country. Eligibility for awards included having less than $7.5 million in total NIH research funding, and at least 25% Pell Grant recipients. The grantees serve a geographically and racially diverse population that include two historically Black colleges and universities, five Hispanic-serving institutions, one of which is also an Asian American/Native American/Pacific Islander-serving institution, and campuses with targeted outreach to special populations that are underrepresented in STEM (see Table 1). Each BUILD-funded site employed different approaches to develop capacity for meeting program goals, including research skill building, training (for students and faculty), and infrastructure development [49]. (For more information about BUILD and the broader Diversity Program Consortium (DPC), see https://www.nigms.nih.gov/training/dpc/pages/build.aspx).

**Table 1:** Profile of BUILD Sites

Institution	Date of Site Visit	Institutional Type and Minority Serving Institution Status	Institutional Characteristics^a^
California State University, Northridge (CSUN)	2016–2017	Public HSI	Undergraduate Enrollment: 36,979 Admission Rate: 48% Pell Grant Recipients: 54%
California State University, Long Beach (CSULB)	2016–2017	Public HSI/AANAPISI	Undergraduate Enrollment: 32,025 Admission Rate: 32% Pell Grant Recipients: 51%
Xavier University	2016–2017	Private HBCU	Undergraduate Enrollment: 2344 Admission Rate: 62% Pell Grant Recipients: 54%
University of Texas, El Paso (UTEP)	2016–2017	Public HSI	Undergraduate Enrollment: 20,004 Admission Rate: 100% Pell Grant Recipients: 57%
Portland State University (PSU)	2016–2017	Public N/A	Undergraduate Enrollment: 19,648 Admission Rate: 89% Pell Grant Recipients: 40%
Morgan State University (MSU)	2017–2018	Public HBCU	Undergraduate Enrollment: 6294 Admission Rate: 60% Pell Grant Recipients: 56%
University of Maryland, Baltimore County (UMBC)	2017–2018	Public N/A	Undergraduate Enrollment: 11,144 Admission Rate: 57% Pell Grant Recipients: 28%
SF BUILD: San Francisco State University (SF State) & University of California, San Francisco (UCSF)	2017–2018	Public // Public N/A // NA	Undergraduate Enrollment: 25,867 Admission Rate: 68% Pell Grant Recipients: 45%
ReBUILD Detroit: Wayne State University (WSU) & University of Detroit Mercy (UDM)	2017–2018	Public HBCU Private Catholic N/A	Undergraduate Enrollment (WSU): 16,728 Admission Rate (WSU): 81% Pell Grant Recipients (WSU): 45% Undergraduate Enrollment (UDM): 11,144 Admission Rate (UDM): 78% Pell Grant Recipients: 29%
University of Alaska, Fairbanks (UAF)	2017–2018	Public N/A	Undergraduate Enrollment: 5432 Admission Rate: 73% Pell Grant Recipients: 21%

*HSI* Hispanic Serving Institution

*HBCU* Historically Black College and/or University

*AANAPISI* Asian American Native American Pacific Islander Serving Institution

^a^Derived from Integrated Postsecondary Education Data System (IPEDS) 2016 Data

### Site visits

From 2016 to 2018, a team of at least four researchers conducted telephone or Zoom interviews and subsequently visited each BUILD site to conduct interviews and observations of the programs. They spoke with BUILD program faculty, and staff and students, as well as institutional leaders (e.g. President, Provost, Vice President for Research, etc.). Working with program PIs or designated BUILD program staff, the study researchers identified and recruited over 30–60 staff and faculty participants across the 10 BUILD grantee sites for individual interviews and focus groups [50]. Informed consent was obtained from all participants in the study prior to conducting interviews, and appropriate interview protocols were developed depending on the participants’ roles (see Supplementary Material). Site participants were asked about goals, progress, challenges, and strategies employed to achieve program initiatives in order to get a general sense of each site’s program implementation. Interviews and focus groups ranged from 45 to 90 min each and were recorded, transcribed, and uploaded to Dedoose qualitative software for analysis. After each site visit, members of the research team met with each site’s primary BUILD program leaders to review a debrief report created by the research team, allowing an opportunity to offer insights and clarify observations made during the site visit. All methods were carried out in accordance to relevant guidelines and regulations approved by the Internal Review Board at the University of California, Los Angeles.

### Coding and initial site analyses

To make sense of the data collected, research team members developed a codebook to account for four embedded levels within the data: institutional-wide, program-specific, faculty-specific, and student-specific. The codebook was created to deductively examine how constructs from the existing literature and the Diversity Program Consortium’s program objectives [51] were present within the data. Codes were also generated inductively to capture unique BUILD processes. Coders primarily included members of the research team that visited each site, as well as members of the broader research team. Coders characterized meaningful patterns across interviews to assess a general sense about “what is going on [at this particular BUILD site]?” ( [52] p. 88) and employed a descriptive coding strategy [53, 54] to summarize the primary topic of excerpts. To assess the extent of agreement among data coders after initial coding [55], an inter-rater reliability test on Dedoose was used and the inter-coder reliability score was .96 across all ratings, based upon a Pooled Kappa [56]. Given the number of codes cross-checked on four levels among coders, the team deemed these ratings as sufficient levels of agreement to proceed with the coding of the remaining transcripts.

The research team also created written debrief reports after each site visit which they shared with PIs and program leaders [50]. Debrief reports contained summaries and general impressions of each campus as related to the case study’s overall research questions. Using the debrief reports and transcripts, members of the research team wrote case narrative reports to summarize themes within the context of each institution [47].

### Cross-case analysis

A multiple case study design can identify similarities in approach, logic, or conditions that inform the phenomenon of interest [47]. We ran data queries using Dedoose to pull specific codes related to institutionalization and program sustainability from all interviews at all institutions. This data was supplemented and contextualized by information from the debrief reports, case narrative reports, BUILD site websites, and articles summarizing each BUILD site’s intended goals and initiatives [14]. To make sense of the large amount of data [57], the research team employed data reduction [53].

The authors then employed second cycle coding techniques [52] such as focused coding [58] and wrote analytic memos to combine individual codes into broader themes and make connections to prior literature on institutionalization and sustainability. Criteria for answering this study’s research question involved looking for early evidence of institutionalization and sustainability, such as participants who mentioned actions that had already taken place to sustain programs, and/or specific plans that would be enacted in the near future. Opinions about the sustainability of a program, as hopes for sustainability without mention of detailed plans for action were excluded. The researchers also focused on triangulating data by paying attention to whether at least one other participant at the site provided a similar response with respect to program sustainability and characteristics that exemplified early institutionalization. After deriving initial themes to explain interviewees’ answers to program sustainability and early institutionalization efforts for each BUILD site, the authors developed matrices, which allow researchers to “see” the data, its frequency and qualitative differences across sites and/or embedded cases [53]. The use of matrices not only helped determine how codes related to each other, but was also a good method to use to make contrasts and comparisons between institutions [53]. When we differed about particular findings for each research question, we reexamined transcripts, matrices, or themes in question and discussed it until consensus was reached [59].

### Positionality

We position ourselves as education researchers of color interested in STEM equity and involved in evaluation of the Diversity Program Consortium, which includes overall evaluation of the BUILD program. We aimed to mitigate any biases by employing strategies such as developing a systematic analysis and audit trail to document steps in thinking and theme development, especially when working with large data [60] in addition to taking measures to ensure trustworthiness and credibility.

### Ensuring trustworthiness

The trustworthiness of findings is important in qualitative research [61, 62]. As an initial form of member checking, meetings were held between the research team and site program administrators following each site visit where representatives from each BUILD site had the opportunity to discuss the research team’s initial impressions. We met as co-authors frequently over the course of 8 months to discuss themes (and their associated coded data). We reorganized and reconfigured themes to eventually develop a smaller and more select list of broad categories, sub-themes, concepts, and/or assertions. To triangulate findings on sustainability, we relied on obtaining convergence from multiple sources of interviews and case study data. As a method of ensuring trustworthiness, we systematically searched for divergent cases that should be included in the analysis and discussed in order to include important nuance to the findings. Finally, research team members who conducted the site visits and had additional knowledge of the sites were asked to review the manuscript to identify any errors and provide feedback regarding themes.

## Findings

BUILD participants reported on strategies to sustain their programs that were occurring simultaneously to implementing, evaluating, changing, and measuring outcomes. Since all these efforts took place concurrently, participants’ perceptions and opinions of program efficacy and the likelihood of sustainability needed to be separated from actual evidence of adapting and embedding aspects of the BUILD program. In light of this, we identified five categories of approaches that BUILD sites employed to *actively* develop sustainability of BUILD efforts during the third or fourth year of implementation of their five-year and potentially 10-year grants.

Overall, we found that BUILD sites actively engaged in developing program sustainability by employing a combination of the following strategies 1) scaling and adapting to expand impact, 2) identifying additional funding and implementing cost-cutting measures, 3) developing and maintaining infrastructure and structural operations, 4) leveraging relationships with intra-and inter-institutional partners, and 5) addressing hiring, policies, and reward systems on campus. The combination of strategies used varied by institutional context: institutions with prior active STEM programs and faculty focused more on scaling and seeking additional funding, while institutions with fewer prior STEM-funded initiatives and research capacity focused on developing infrastructure and structural operations as well as changing policies and reward systems. Institutional support from senior administrators, and efforts that included culture change with structural change were related to program sustainability efforts. Finally, while our analysis primarily focused on early efforts of program sustainability, we also found evidence of early institutionalization and divergent strategies that focused primarily on culture change as an approach to achieve program sustainability. We expand upon these themes in the sections that follow.

### Scaling and adapting to expand impact

A common strategy was to take a successful program element and looks for ways to scale up or adapt into larger campus programs and initiatives in the early implementation years (year 3–4). Almost all BUILD sites could identify at least one innovation on campus with evidence of success and enough resources on campus to either scale up and increase the number of students and/or faculty impacted, or adapt into a repackaged format that had the potential to achieve the same outcomes. Several sites were beginning to see success with summer research programs, research courses developed specifically for BUILD-affiliated students, and mentorship training sessions for biomedical faculty. In particular, some sites expressed that expanding exposure to undergraduate research opportunities was a major goal where efforts were being directed, but only a few were already at the stage of deciding how to make these opportunities sustainable. One of the administrators at Morgan State University (MSU) elaborated on the process and rationale to employ the strategy of expanding their entrepreneurial-focused research sequence:

Everything has been developed to a point that we want to make that a two semester course … regardless, even if we are not funded, we want to make that [BUILD research course sequence] university-wide … we think that is good because students have liked it, their mentors have liked it, near-peer mentors have liked it, and now we have evaluation results... we want to make it a course so that it becomes sustainable regardless of large funding.

While they had not yet reached the stage of actually scaling, they had a detailed plan that would easily make this next step toward sustainability a reality. Similarly, California State University, Long Beach (CSULB) was in the process of expanding curriculum through interdisciplinary efforts involving four STEM colleges on campus under an umbrella health-related curriculum that addresses health disparities, and is the first of its kind at CSULB to include faculty and students from such a wide range of departments. A senior administrator noted:

Each college has their specific equipment, facilities and so forth … Often, those centers are geared toward supporting their own students and faculty. Now, because four colleges are working together, all those centers and institutes are open to, not only their own students, but students from other colleges as well. That's huge.

Although CSULB reported bureaucratic challenges, they saw that their campus culture was shifting toward valuing the interdisciplinary, health-centered approach to achieve goals of increasing the number of underrepresented students in biomedical fields, and thus began trying to sustain this institutional shift by expanding efforts to impact all four colleges.

Another strategy was to focus on sustainability for every element of the BUILD program from the beginning. For example, one program administrator at University of Maryland, Baltimore County (UMBC) noted that their goal was to expand the program to become part of their usual way of operating, “One of our core PIs is constantly saying to us, ‘Think 500 not 50.’ I would say that we have really had sustainability and scalability in the forefront of everything that we’ve done since day one.” UMBC focused on making changes that could be financially sustainable, rather than the approach of using funds to pay for the operating expenses of new innovations. UMBC program coordinators reported that program elements such as the STEM living learning community (acquiring dorm space and adding a student affairs staff member), student advising model, and undergraduate research would be sustained because models for these already existed at the institution and/or required little investment to expand and adapt to reach more students.

### Identifying additional funding and cost-cutting

In the fourth year of funding, the NIH program officers asked sites to think about how to gradually reduce dependence on grant funds each year as the programs matured. Interviews with case study participants show how sites were strategizing around budgets. Strategies mainly focused on seeking new funding for student stipends and undergraduate research experiences, resources to run developed initiatives, as well as finding ways to run programs with reduced funding.

Stipends provided to students engaged in the BUILD programs were often cited by participants as the most expensive part of program implementation, but a necessary part of recruitment and engagement. Many participants believed that these stipends were critical since underrepresented students’ capacity to engage in scientific skill-building engagement activities within BUILD programs depended on students’ financial resources. A few sites that were trying to sustain scholarship funding mentioned seeking private donors through campus development efforts. However, the level of ease of raising funds appears to vary by biomedical discipline and support of senior leadership. A college of engineering administrator at ReBUILD Detroit mentioned:

We continue to try to solicit funding to continue [funding for biomedical-related support, and that is part of my job, through donors or through foundations and federal grants. I would say we probably rely more heavily on our donors, not exclusively, but I would say more so. And I would say we've had, historically, more success on the engineering side than the science side, just in engaging our alumni and developing them into donors.

Developing faculty capacity to apply for research and training grants was a component of all BUILD awards, and several campuses noted their plans to continue raising additional funding for research grants that would provide faculty the means to mentor and engage biomedical students. PSU, for example, worked on a NIH RISE proposal, which is “going to be basically a pathway for the [BUILD] scholars to get into Oregon Health and Science University.” A senior administrator said the proposal focused on linking their undergraduates with graduate education to “make sure that we don’t let the momentum that has started from the [BUILD] grant” subside. While a lot of BUILD research experience initially occurred at research-intensive partner sites, grant-funded research projects obtained by a site’s faculty would allow for undergraduate research training and mentoring to continue on these campuses, in addition to providing career development and CV-building for the faculty involved in the grant-writing/awards. The challenge of this strategy is that additional efforts must also be invested in training faculty to submit grant proposals, in addition to having willing faculty who are interested in taking on the added responsibility of running a research project and mentoring student trainees. Participants mentioned the challenge of this goal, especially at primarily undergraduate teaching institutions where faculty have high teaching loads and are often evaluated on their teaching rather than their research. However, many of the sites were beginning to shift policies and reward systems to support faculty research projects, which we discuss further in the section on shifting hiring, policies, and rewards*.*

MSU invested BUILD funding into the development of the Student Research Center (SRC) and hiring a staff coordinator to support the initiative. The student-led and student-run center is open to all undergraduate full-time students with a grade point average of 2.75 or greater. The SRC has a dedicated renovated suite (initially supported by BUILD funds) in the science complex that is a student lounge and meeting space with state-of-the-art technology. Since the SRC is technically registered as a student organization, the salary for the coordinator of the Center will transition from grant funding to student fees. For other program costs, one administrator reported:

Our Student Research Center … is something that definitely will stay on campus … but it's going to require funding. One of the things that I suggested is that they're going to have to raise funds, like any student organization. There needs to be funding raised in order to maintain some activities that the organization wants to do, and so I think that they have a plan to do some of that.

We also found examples of how sites were simply trying to find ways to reduce costs to continue current programs without changing the structure of the program. UTEP had invested grant funding into developing research-driven courses (RDCs) for first year students, and planned to reduce future costs associated with the courses (e.g., modifying experiments to use cheaper reagents, ordering products in bulk). As a faculty lead of one RDC at UTEP explained:

I think the reality is these courses cost a heck of a lot more money up front to set up than they do to maintain...It won't cost that much to maintain four or five of these as long as we've paid those significant costs upfront to develop that infrastructure to run them … A traditional biology lab is given a TA already. The idea is that these RDCs ultimately will be replacing the traditional biology lab and they would get a TA.

While the original RDCs involved initial funding to set up, and BUILD financial support for postdoctoral scholars and graduate students to support the effort, they can be converted to self-sustaining courses. The faculty lead also explained the plan to approach the Office of Research and Sponsored Projects and the Provost to step up and provide sustainable funding to pay for supplies for the RDCs.

For the University of Alaska, Fairbanks (UAF) BUILD site, the program evolved in response to evaluation data to build the sustainability of a mentoring component of their program. A program administrator shares the decision process that led to shifting from funding graduate students to developing a new position to mentor and support biomedical undergraduates:

We had a program called the graduate mentoring research associates … These were graduate students whose job was to directly work with research for undergraduates... It turned out that when we did the analysis on this, the return on investment, so to speak, was not all we had wanted … we were only getting three or four undergraduates per graduate student. [It] was much more effective to go to what we now call our [Research Advising and Mentoring Professionals] RAMPs. So we negotiated this with NIH and with our team and said, “Look, we're going to drop out these graduate mentorship programs in the future. Instead, we're going to put money into the RAMPs and we're going to put money into exporting RAMPs to these other institutions across the United States.

Through re-evaluation of innovative practices while considering sustainability, UAF administrators ended up with a financially sustainable innovation that they believed enhanced the program’s impact, thus sustaining and even improving desired outcomes.

In sum, many BUILD sites began the process of securing funding from their institutions or via fundraising to maintain initiatives with promising outcomes, or explored ways to shift program elements and cut costs of the activities.

### Developing and maintaining infrastructure and structural operations

Another strategy for sustainability focused on investing funding into permanent structures to serve biomedical training and research, or examining current organization and structures of programs and offices and restructuring in order to enhance student support. All sites invested grant funds toward building infrastructure such as active learning classrooms, research centers, and labs and lab equipment. Sites were finding ways to maintain staff support and/or coordination needed to operate newly-created centers, classrooms, and buildings. For example, one program staff member at Xavier University discussed the mid-grant strategy of restructuring their centers to maintain career development and other components of BUILD:

When we first had BUILD, we had a Career Advancement Center for our Office of Career Services, we also in addition to the Center for Undergraduate Research we have a graduate placement office. It's almost like the parallel of a career services, but it's for students who want to go to graduate school. The Center for Undergraduate Research was separate from those two … When you really look at [the work of both centers], there's a lot of overlap … . We combined the Center for Undergraduate Research and Graduate Placement into one thing. It's now a huge name. It's the Center for Undergraduate Research and Graduate Opportunities. We're calling it CURGO. [Administrator] is the Senior Director of the whole thing.

Rather than attempting to receive funding for multiple offices that support students that want to pursue graduate studies, they evaluated the duties of the different offices, addressed places where overlap existed, and consequently consolidated the offices to promote sustainability of services. The consolidation strategy not only appears to minimize the amount of overlap between different duties, but also promotes sustainability through limiting the amount of funding needed for personnel of different offices while continuing to achieve goals of serving students.

### Leveraging relationships with intra- and inter-institutional partners

#### Inter-institutional efforts

Maintaining successful partnerships with pipeline institutions such as high schools and community colleges, as well as with research-intensive institutions where students could gain undergraduate research experience and potentially go to graduate school, were seen as important to sustaining the impacts of BUILD. Participants mentioned the importance of continuing work with community partners due to the synergy of sharing information/skills, resources, and connecting students. PSU developed unique partnerships with community colleges in the Pacific Rim in an effort to support Pacific Islander students’ biomedical career pathways by mitigating challenges to transferring to a four-year institution. For example, Xavier University developed partnerships with research institutions, such as X and Y, to connect their primarily Black biomedical undergraduate students in BUILD with biomedical research opportunities. Some participants shared that the relationships between faculty and staff, organized through the BUILD partnerships were valuable and sustainable beyond the grant. An administrator at SFSU elaborates:

My feeling is that all will stay in place once the funding is gone. The partnerships between the two institutions, UCSF and SFSU, those [relationships] are stronger. Those connections - once people have met collaborators and worked together on projects, that doesn't change. This year, actually, UCSF accepted more graduate students from San Francisco State than any prior year... The fact that these students are also entering these institutions with a background from San Francisco State, and the curriculum that the SF BUILD put out there, rolled out there, that goes with them wherever they go.

#### Intra-institutional efforts

In addition to partnerships with other institutions, some campuses took the approach of building program sustainability through connecting STEM initiatives on campus to build networks and foster synergy between people, budgets, and events. A senior administrator at PSU shared how the BUILD grant was the catalyst to aligning all STEM programs on campus, and how they were trying to build sustainability of all efforts to support students in STEM by co-locating services and people, as well as pooling together space and financial resources:

I think we have risen to the challenge with all of our various support structures. We actually have co-located our STEM initiative with the [BUILD] EXITO folks all on the same physical location that our office helped to do the renovations for in this minor way. We have a HHMI Howard Hughes Medical Institute grant in the same physical location ...We tried to put all of those types of things [together] ... and the McNair undergraduate program. We have multiple [STEM] programs relating to diversity in higher education in the same physical location.... I think that's going well. [BUILD] was kind of an instigator of [thinking], “how do we take all of these programs that we're doing together and basically have some efficiencies of space?” and try to give [people] opportunities to interact with others outside of their own program.

Leveraging relationships with other on-campus programs fostered efficient use of physical resources and knowledge-sharing in order to sustain program goals for all involved. Additionally, newly-created partnerships may also be evidence of the institutionalization process, as structural changes in knowledge- and resource-sharing become embedded into the institutions.

### Addressing hiring, policies, and reward systems

Hiring diverse biomedical faculty, policy changes, and rewarding faculty for shifting behaviors were also important for sustaining changes initiated by the BUILD program. A few campuses noted the importance of faculty recruitment and hiring as part of institutionalization or permanent changes to increase visibility of diverse faculty, and to have more faculty who can teach, mentor, and conduct research training in ways that align with the goals of the BUILD program. An administrator at UDM noted:

One of the things that we have to work on as an institution is our diversity of our faculty … there aren't many private institutions, particularly those who are Catholic and that have faculty unions … many people stay on a lot longer in their careers than they might in another situation. We want to bring in younger faculty members, we want to bring in more diverse faculty members … when we do hire, we hire and look for some of that diversity within our different departments. Students are attracted to institutions where they know that there is the same kind of diversity that they expect among the student body. That's going to be one of our challenges going forward and one that we have begun to work on.

Commitment from leadership, in addition to precise insight of the challenges to hiring diverse faculty within UDM’s institution’s unique context are both critical to the development of solutions to achieve this goal. Some sites were already looking to the future by hiring faculty whose values aligned with BUILD goals. One campus, CSUN, was aiming to finalize the approval of a health sciences faculty cluster hire. The goal of the cluster hire was twofold: to invest in an interdisciplinary group of faculty who aligned with BUILD goals to increase the diversity of students entering the biomedical workforce, and second, to hire research-oriented faculty who would have a reduced teaching load in order to focus on obtaining grants in their first few years hired on campus. As explained by a program staff member at CSUN:

There is a lot of evidence that cluster hiring does help support increasing diversity … before cluster hiring, our campus had a real push to diversify the faculty … the main goal of cluster hiring is to increase the research activity, so that's our focus. We want to bring in top-notch researchers …

Thus, while the context at CSUN already had aspirational values that supported the hiring of diverse faculty, the sustainability of research training required that BUILD focus on hiring diverse faculty who would be able to support the research infrastructural development and training of underrepresented biomedical students.

Aside from goals to hire more diverse biomedical faculty who reflect student demographics, sites also focused on updating policies, evaluations for tenure and promotion, and creating rewards in order to reflect new shifts toward values of mentoring, improving pedagogy in biomedical disciplines, and conducting biomedical research. Many sites identified faculty mentor training programs as important to continue due to varying levels of success in shifting culture on their campuses. Some sites found funds to incentivize faculty to attend mentor and other training and made it required as part of new faculty orientation. Xavier took a unique approach by requiring mentor training for any faculty who apply for research grants with plans to include student researchers:

Now, on the proposal clearance form that faculty have to use when they are doing a routing for their [research] proposals, there is a question that asks [the faculty member] if they have gone through mentor training. If they have put [a] budget in their grants for students, we expect them to go through mentor training because they need to serve as good mentors to these students.

Strategies focused on changing policies to incentivize and reward behaviors that support biomedical training of underrepresented groups (e.g. culturally aware mentorship) are evidence of the institutionalization process. Over time, these practices can become typical expectations for faculty activity at the institution.

### The impact of senior administrative-level support on building program sustainability

Involvement of senior administrators (e.g. presidents, provosts, deans) and faculty in leadership roles was critical to sustainability efforts because senior leaders were able to prioritize the program’s goals and redirect resources in order to sustain program elements and/or begin the process of embedding program goals into the institution’s operations.

At a few of the institutions where senior administrators were part of obtaining the BUILD grant and were involved from the start led to additional investments to support BUILD efforts. For example, the president at WSU (one of the institutional partners of ReBUILD Detroit) strategically invested $400,000 in the BUILD program, making ReBUILD Detroit the only program where the research-intensive partner completely self-fund its commitments to BUILD. This funding enabled the campus to operate with greater autonomy and flexibility to achieve program goals. It is important to note that the WSU President had previous experience working at NIH (and as a former advisor to the Diversity Program Consortium (DPC) Steering Committee). Thus, the President’s work on diversity initiatives carried over into his leadership of WSU. At UAF, the paradigm that guides the BUILD program—the interdisciplinary One Health Model [63]—is financially supported by senior leadership and fully embedded into the institution with future plans for driving research, curriculum, and student engagement.

At other sites, PIs worked to involve senior administrators after they received funding. Challenges to this effort included changes in leadership over time at various institutions. However, what is important is that support from senior administrators was typically needed to obtain institutional funding, in addition to support for the other strategies that we identified for actively building sustainability. A program administrator at CSULB explains the involvement of the upper administration:

I meet regularly with the [campus administrator routinely], but also for BUILD because … he has the responsibility for that, too. In fact, it was interesting when they hired our new [campus administrator], in the (position) description, it was written that part of his new responsibility was the BUILD award.

The administration’s responsibility to BUILD is further solidified by one of BUILD’s leaders who stepped out of her position as a campus administrator to work on BUILD initiatives full-time. As a part of her focused engagement, she brought her long-standing cross-campus connections, clout, and a practical understanding of the administrative processes in the institution to sustain program implementation.

### Sustainability without institutionalization

While institutions with leadership support were able to more easily build and plan for sustainability and active institutionalization of innovations, campuses without institutional leadership support tried to infuse their program throughout the campus in other ways. At one institution a faculty member shared progress made with faculty Critical Race Theory (CRT) training, “As far as faculty go, we already have about a hundred faculty on board, out of the 650 full-time … through faculty affairs, that’s where we were planning to expand the idea of CRT through that mentor training that they do.” This strategy involves garnering wide-spread faculty involvement that will help to sustain initiatives through faculty development in mentor training.

Another faculty member at the same institution alluded to how upper level administrative support could unleash the potential of dedicated faculty and staff to support underrepresented students in STEM:

I would say it's sustainable because we have people like [faculty and staff colleagues] … who are willing to do the work and are doing the work anyway. Whether or not BUILD is here, they're going to be doing the work. Whether or not they're paid to do it, whether or not they're getting recognition … we are all very invested in these communities. We're not talking about abstract concepts or objects, this is our family … This is everything to us, so this is going to get done by these people whether or not it becomes institutionalized. I can only dream and imagine the work of [a BUILD colleague] and all these people could possibly do if they had the institutional support. If it came down from the president, from the provost saying, "Go for it. Let's do it."

At this institution, key change agents were present and invested in STEM equity, however a challenge on their campus in the early stages was obtaining top level administrative support to formalize and institutionalize progress made with the current initiatives. This case underscores how conceptions of sustainability and institutionalization may differ. At this institution, sustainability might include changing faculty mindsets and behaviors from the ground up, resulting in sustained changes at the faculty level without formal institutionalization. Support from senior-level leadership may be include financial support and/or prioritization of faculty training that can further embed this program element into fully supported part of the institution.

A few participants emphasized that cultural change was an intentional strategy that they believed was critical to long-term program sustainability (i.e. focused on changing individual behaviors of biomedical faculty and students). Specifically, they believed that espoused values embedded in BUILD initiatives could be passed onto faculty, administrators, and students in order to change behaviors, and ultimately shift the culture of the campus. Thus, even when grant funding ends and many of the created programs will no longer exist, some participants expressed that knowledge left from any training and short-term efforts could maintain a culture that achieves the program goals of increasing diverse participation of researchers in the biomedical disciplines. For example, the SF BUILD site developed stereotype threat training modules in order to familiarize administrators, mentors, students, and faculty with a model that facilitates diversity in the biomedical sciences. One participant described the initiative spreading across campus:

There was initiation of thinking about ways of creating a more welcoming environment … SF BUILD program then provided additional levels of training and awareness that then spread across the college … So it wasn't just in biology, but now these discussions were being held in math and psychology, and to some degree, chemistry and biochemistry.

Whether or not there were tangible plans to continue offering the stereotype training, participants believed that changed values and behaviors would be self-sustaining. Another example of efforts focused on changing values of individuals involved in biomedical education comes from a faculty member at CSUN:

Just thinking about training and trickle-down effects … junior faculty many times are looking for mentorship from senior faculty, and when we see senior faculty that are successful in terms of their program of research or that are offering guidance in terms of issues pertaining to [BUILD site’s theoretical framework], I think it creates that continuation of receiving that mentorship from above and then providing that mentorship to your own students. [Those are] aspects that I could see in terms of being able to sustain progress.

The belief that changing values and mindsets “trickle down” as a way of sustaining progress of the BUILD program illustrates how some participants conceptualized sustainability without formal institutionalization. Similarly, a ReBUILD Detroit administrator shared:

I think the advances that our faculty, the skills that they've learned, in terms of mentoring, in terms of doing research, they will have that now. That will help them for the rest of their career. So I think that that is there. We've learned things that don't have anything to do with continuing funding.

A few participants believed that learning that occurred from even one-time offerings of training and workshops, regardless of whether they continued to be offered on campus to additional staff and faculty, was evidence of sustainability. These divergent approaches to sustainability hold promise if engaged faculty have actually changed their behaviors and are a stable part of an institution. A focus on development and training requires that faculty and administrators who have received training actually change their values and behaviors. Additionally, because faculty and staff members who have received training leave an institution sooner or later, it is less likely that the changes would be self-sustaining in the absence continued offerings of training and workshops for newly hired staff and to serve as a booster for already-trained staff.

## Discussion

This study sought to understand how campuses were building program sustainability and early stages of institutionalization of initiatives that have promise in advancing program goals. We captured a snapshot of the state of BUILD sites, during their third and fourth years of program implementation, prior to knowing whether or not they would receive a five-year renewal of NIH funding. Although site participants often used terms like institutionalization, sustainability, and culture change interchangeably to explain their early efforts to build ways to sustain programs under development and implementation, their different approaches aligned with the concepts in the literature that support sustainability and characteristics of institutionalization as a process [7]. We found BUILD sites engaged in the following strategies to develop program sustainability and begin to institutionalize BUILD initiatives: 1) scaling and adapting to expand impact, 2) identifying additional funding and cost-cutting measures, 3) developing and maintaining infrastructure and structural operations, 4) leveraging relationships and with intra-and inter-institutional partners, and 5) addressing hiring, policies, and reward systems. We also identified situations where participants felt that they were on track for sustaining BUILD program elements (e.g., finding other funding sources to continue some aspects of the program) with less focus on embedding program goals, practices, and outcomes into the institution. These processes extend the notion that institutionalization is not simply about moving from grant funding toward institutional funding but rather a process of embedding structural and cultural change into the institution.

Our analysis revealed lessons for moving grant-funded initiatives into regular campus programming and infrastructure. First, we found early evidence of institutionalization processes at many BUILD sites. Specifically, some BUILD sites were attempting to shift the organizational culture on their campuses, create structural and cultural changes, and consider which aspects of their program could be realistically sustained by examining financial, physical, and human resources unique to their contexts. Evidence of early steps towards institutionalization include infrastructure and organizational structural changes to increase biomedical research capacity, new support services for biomedical students, and enhanced biomedical curriculum. In particular, the literature on institutionalization highlights the importance of building organizational infrastructure in order to embed a new innovation [33]. However, some of the BUILD programs also strived for sustainability without formalized institutionalization [44]. This study shows how some campuses strive for sustainability in ways that also involve institutionalization processes.

Second, actively building sustainability and/or institutionalization is an iterative process, especially in the midst of implementation as program teams problem-solve issues that arise. Adapting and changing is necessary as an institution evaluates what works and how to best sustain the physical and cultural changes to a campus. For example, UAF’s original program that relied on graduate student mentors had to change when the evaluation showed that the model was not financially sustainable, resulting in a new, more flexible and fundable staff position (RAMPS). The intention of UAF to achieve and sustain goals, represents sustainability, but has yet to show institutionalization of these positions over the long term. Although, in combination with UAF’s financial support from senior leadership as a result of the One Health model, UAF began to extend initial research and curriculum initiatives across the institution that made infrastructure consistent with their context and rural/indigenous culture [63].

Third, this analysis suggests that program sustainability may be possible without institutionalization, however, institutionalization may not be possible without program sustainability. For example, some sites were going to continue to support their initiatives with other new grants, rather than obtaining institutional funds. Thus, programs could be financially sustained, but not structurally supported by the institution via financial investment. While some BUILD sites discussed sustainability as the hope of changing the mindsets and behaviors of colleagues involved in biomedical research training, institutionalization requires wider institutional buy-in and accountability. The challenge of one-time training for faculty development aimed at changing practices is that change is unlikely to be sustained nor institutionalized due to turnover of faculty, staff, and leadership. Further, research indicates that those with a more extensive faculty development history show measurably larger changes in their behaviors (e.g. teaching methods) than faculty whose participation is slight, such as a single department workshop [64].

Fourth, we found evidence to support prior findings regarding the importance of leadership [5] in order to support or be a catalyst for engaging in institutional transformation. For example, the presidents at WSU and UAF provided financial support and made BUILD a priority, providing funding and additional status and influence on campus for the initiatives.

Our research also provides clarity to the organizational change literature on sustainability and institutionalization processes and strategies employed by STEM program administrators; this is important insofar as we provide context for how these concepts interact in the field, something not extensive in prior research. Consequently, our research will provide grant-funded initiatives in STEM examples of how they can embed their program structures and values into the institution for advancing underrepresented student success.

### Limitations

There are several limitations to this present study. First, the research team consisted of several members with changes in group membership during various phases of data collection and coding. As a result, various team members had different working knowledge and familiarity with each BUILD sites’ program elements and progress on program implementation. To address this issue, multiple meetings were held throughout the year between incoming and continuing team members in order to establish continuity of knowledge and team organization [65]. Second, site visits were conducted in two phases, such that five of the campus site visits were conducted within the third year of a 5-year grant implementation cycle, and the other five site visits conducted in the fourth year of implementation. By the time the last round of institutions were visited, they had already geared up to write for renewal of their BUILD grants. In other words, the sites visited in the second phase had already begun to think more systematically about program sustainability, although the extent to which sustainability efforts were effective in measurably improving biomedical diversity goals is beyond the scope of the present study. Hence, in our analysis, we were mindful of the fact that the later site visits would naturally have more to share regarding efforts to build sustainability. While one of the goals of the BUILD initiative is to promote institutional change, we focused more on what sites were doing early on to create self-sustaining components as they were implementing their grant-funded program.

## Conclusion and implications for research, theory, and practice

Overall, this study provides evidence of distinctions and interrelations between program sustainability efforts and institutionalization of change efforts. We found that program sustainability strategies can overlap with the process of institutionalization, especially strategies that focus on organizational and infrastructural change. When campuses enter the final years of this 10-year NIH initiative, research can assess how the early efforts in creating sustainable programs contributes to institutionalization of innovations to enhance diverse participation in biomedical education and career training. Researchers can also examine the sites that employed the sustainability strategies that overlapped with the concept of institutionalization to determine whether or not their strategies support or begin to modify theories about creating lasting institutional change. For example, CSUN provides an example in the form of attempting to change the culture via faculty mindsets [40] via race and cultural awareness training, but widespread buy-in and adoption depends on upper level leadership support, and integration of critical mentorship activity into rewards for tenure and promotion, which in turn may present a challenge to the promise of real institutional change. Not all grants funders nor awardees may have the same goals of institutionalization, so clarifying and setting common goals for both grant funders and grantees can increase the chances of meeting expectations to achieve institutionalization. Some campuses may attempt to sustain BUILD teaching and learning strategies, while others are attempting to create lasting change (i.e. institutionalization). Future research can distinguish these objectives. Future research can also continue to explore how sustainability and institutionalization are achieved, and how such strategies are interrelated or distinct. While some may conceptualize institutionalization and program sustainability as interchangeable concepts, strategies for sustainability after grant funding has ended is different from the strategies used to achieve institutionalization (i.e. embedding practices in the institutional culture). Program sustainability might be process-oriented (e.g., securing resources to continue a project) or outcome-oriented (e.g., a project ended because its goals have now shifted the culture of the institution or combined with other institutional initiatives). Process-oriented sustainability can be obtained without institutionalization processes, but outcome-oriented sustainability will be easier to with an approach that considers ways to embed the program into the fabric of the institution. Additionally, future research can also focus on metrics for success of program outcomes alongside metrics for program sustainability and institutionalization. Otherwise, gains that result from long-term initiatives like BUILD could be limited to the timeframe of the initiative.

For principal investigators of STEM program grants and institutional leaders, findings indicate the importance of developing early sustainability plans that lead to institutionalization. Constant evaluation of both the efficacy of programs and efforts toward sustaining successful aspects of programs can support early institutionalization.

For grant funders, including a sustainability plan along with their objectives for institutionalization in RFAs, and providing awardees with resources and suggestions to build sustainability can help mitigate barriers to institutionalization. This study begins to provide needed guidance for these projects. For example, funders can ask questions and provide guidelines for how institutions plan on embedding changes in culture and practices, (e.g. in HHMI’s new Driving Change initiative) [66] or simply ask for a plan to sustain the proposed programs. Finally, funder workshops that provide research insights on organizational change, sustainability, and institutionalization may increase program administrators’ awareness, knowledge, and sustainability planning. Future research on BUILD’s next phase of program implementation will reveal both the success of sustainability, and ultimately institutionalization, as well as well as success in achieving the program objectives adopted by all members of the Diversity Program Consortium.

## Supplementary Information

**Additional file 1.** Semi-structured Case Study Protocols.

## Data Availability

The data that support the findings of this study are available from the Coordination and Evaluation Center (CEC), but restrictions apply to the availability of these data, which were used under license for the current study, and so are not publicly available. Data are however available from the authors upon reasonable request and with permission of CEC.

## Abbreviations

BUILD: Building Infrastructure Leading to Diversity
STEM: Science, technology, engineering, and mathematics
