# Supplementary material for: A cross-case analysis of developing program sustainability and institutionalization in early stages of a multisite biomedical student diversity initiative
Source: BMC Med Educ. 2021 May 3;21:254. doi: 10.1186/s12909-021-02663-2 (PMC8091701; doi:10.1186/s12909-021-02663-2)
Supplement: Supplementary file 1 — Additional file 1. Semi-structured Case Study Protocols. [file 12909_2021_2663_MOESM1_ESM.docx]

**A Cross-Case Analysis of Developing Program Sustainability and Institutionalization in Early Stages of a Multisite Biomedical Student Diversity Initiative**

Authors:

Krystle Palma Cobian^1,^ Hector V. Ramos^2^

1. Fielding School of Public Health, University of California, Los Angeles

1100 Glendon Ave, Suite 710, Los Angeles, CA, 90025, USA

1. School of Education and Information Studies, University of California, Los Angeles
   1. Portola Plaza, Los Angeles, CA, 90095, USA

§ Corresponding author:

cobiank@ucla.edu – 1100 Glendon Ave, Suite 710, Los Angeles, CA, 90025, USA

**Declarations**

**Ethics Approval and Consent to Participate**

This study is approved by the University of California, Los Angeles IRB#15-002023. All methods were carried out in accordance with relevant guidelines and regulations. Informed consent was obtained from all participants whose data are used in this study.

**Consent for Publication**

N/A

**Availability of Data and Materials**

The data that support the findings of this study are available from the Coordination and Evaluation Center (CEC), but restrictions apply to the availability of these data, which were used under license for the current study, and so are not publicly available. Data are however available from the authors upon reasonable request and with permission of CEC.

**Competing Interests**

The authors declare that they have no competing interests.

**Funding**

Work reported in this publication was supported by the National Institutes of Health Common

Fund and Office of Scientific Workforce Diversity (USA) by U54GM119024 and U54GM119024-03-S1 administered by the National Institute of General Medical Sciences (NIGMS). The content is solely the responsibility of the authors and does not necessarily represent the official views of the NIH.

**Authors’ contributions**

K.P.C. contributed to the framing and conclusion of the manuscript. Both the K.P.C. and H.V.R. conducted the data analysis and wrote the majority of the manuscript.

**Acknowledgements**

We thank several members of the Coordination and Evaluation Center who contributed to data collection, preliminary coding, and/or manuscript review: Nicky MacCalla, Dawn Purnell, Jennifer Ho, Damani White-Lewis, Michael Moses, Sylvia Hurtado, Lourdes Guerrero, and Steven Wallace. We also thank Emerald Nguyen and Kenneth Gibbs for feedback on an earlier version of the manuscript.

**Supplementary Material**

**Semi-Structured Protocol - Principal Investigators & Project Directors**

Please state your name and role for the record, and how long you have been at institution name in this capacity.

1. **[Context of current BUILD model & design]** In Year 3 of the program, what aspects of the activities under [the XXX Core/BUILD program], in your opinion, really stand out in their implementation? Why?
2. **[Identifying innovations]** Of these, to what extent do you feel that these reflect innovative approaches to increasing diversity in the population of students pursuing biomedical research careers? Why?
3. **[Strategies to promote diversity]** In your view, to what extent is [the XXX Core/BUILD program] reaching who it is intended to reach?
   1. How is [XXX Core/BUILD program] responding to the need to diversify the population of students pursuing biomedical research careers?
   2. **Probes:** Layers of participants - Students, faculty, mentors, mentees. Who is not being reached? Perceived supports and challenges?
4. **[Strategies to promote diversity]** To what extent has [BUILD program] made alterations in the way it is implemented to better meet the needs of its target participants? [Probe for specific examples]
   1. **Probe:** How may this have affected the diversity of students pursuing biomedical research careers?
5. **[Institutional climate]** In your opinion, to what extent do major players within your institution understand, or buy into the objectives of [BUILD program] and why? Who are those major players? [Probe for specific examples]
   1. **[Institutional change]** What strategies, if any, have you employed to increase institution-wide support of [BUILD program]?
      1. **[Impact]** To what extent do you think these have contributed to the overall success of [BUILD program]? Why?
   2. **[Institutional change]** What might be some of the challenges you face in gaining traction within your institution? [Probe: for students, for faculty, for higher administration]

**Sustainability & Partnerships**

1. **[Sustainability needs]** What are, or will be the necessary supports required to promote sustainability of [BUILD program]?
2. **[Challenges to sustainability]** What, if any, challenges are there in obtaining those supports?
3. **[Partnerships - expectations]** What are your expectations for partnerships?
4. **[Partnerships - benefits]** In your view, which, if any partnerships have most supported the efforts of [BUILD program]? How so?
5. **[Partnerships - challenges]** In your opinion, in what ways might your partnerships pose challenges either to the successful implementation of [BUILD program], or to its success in meeting its objectives?

**Study Visit Preparation**

1. As we prepare for our study visit, is there anything you feel would be important for us to ask of students and/or faculty?
2. Is there anything we should keep in mind as we observe ongoing activity?

**Semi-Structured Protocol - BUILD Faculty – Leads/Co-Leads/Participants**

Please state your name and role for the record, and how long you have been at institution name in this capacity.

1. Please describe what role you play in implementing [BUILD program] activities, and how long you have been supporting the program in this position.
2. How did you come to be involved with [BUILD program]? Why?

**Innovations in Student Diversity, Engagement & Retention**

1. In your opinion, to what extent do you feel the BUILD activities you oversee/participate in enhance diverse student participation and engagement in bio-medical research? [probe for specific examples]
   1. **[Innovation]** What aspects of your program do you believe are particularly innovative in attracting, and keeping a diversity of students interested in bio-medical research?
   2. **[If mentoring is mentioned]:**
      1. What about your current programming elicits student and faculty engagement and participation in mentoring?
      2. From your perspective, what should be the priority areas of focus of mentor training and development?
      3. To what extent do you feel that NRMN programming has contributed to BUILD’s capacity to advance URM bio-medical research training? Areas for improvement regarding NRMN engagement?
2. What impediments might exist to promoting greater diversity in student engagement and participation?
   1. To what extent have these challenges been addressed?

**Strategies to Increase Student Diversity**

1. **[Student composition]** How would you describe the students you engage with under BUILD?
2. **[URG needs]** In terms of enhancing bio-medical research training for URGs what, in your perspective, student needs are the highest priority at this time?
   - 1. To what extent do you feel [BUILD program] is meeting those needs?

**Strategies to Promote Faculty Development & Engagement**

1. **[Professional development]** Have you participated in any [BUILD program] faculty development activities? If so:
   1. Which?
   2. To what extent do you feel they were beneficial to you?
   3. In what ways do you feel these activities could better support your own professional development?
2. **[Faculty buy-in to BUILD]** From your perspective, what about your current programming elicits faculty engagement and participation in BUILD programming?
   1. What impediments might exist to greater faculty engagement and participation?
3. **[Meeting URG needs]** In terms of enhancing bio-medical research training for URGs what, in your perspective, faculty development needs are the highest priority at this time?
   1. To what extent do you feel [BUILD program] is meeting those needs?

**Institutional Change**

1. **[Institutional climate]** How would you say BUILD activities "fit" into the larger vision of [*site name's*] work in bio-medical research?
2. **[Institutional supports & challenges]** To what extent do you feel institutional structures, systems, or processes are in place to fully support [BUILD program]? Why?
   1. To what extent does this level of support differ from that offered by your institution prior to the introduction of [BUILD program]? [probe for specific examples]
   2. What additional supports, systems, or policies do you feel would contribute to better outcomes for [BUILD program]? Why?

**Sustainability**

1. **[Sustainability needs]** What are, or will be the necessary structures, systems, or processes required to promote sustainability of [BUILD program]?
2. **[Challenges to sustainability]** What, if any, challenges are there in obtaining those supports?
3. Is there anything I did not ask about but you want to share about this campus and its role in expanding and diversifying the biomedical workforce? (e.g. Achievements, developments, vision)

**Semi-Structured Protocol - Provosts’- Chief Academic Officer Protocol**

State your name and role for the record, and how long you have been at institution name in this capacity.

1. **[Impact]** NIH has made the decision to invest in institutions instead of only intervention programs. A large investment of xx million dollars was made in this campus to advance the progress and diversify the biomedical research workforce. From your perspective, what has been the impact on this campus of this investment? (Institution-wide changes, recognition, synergy with campus- planning for example).
2. **[Advancing Student Success]** Given the diversity of students and the campus context, how has the campus been engaged in improving the diversity of students graduating and moving into biomedical research careers? What remain key challenges?
3. **[Advancing Faculty Research]** This NIH initiative has a focus on improving faculty opportunities for research, advancing curricular change, and developing faculty capacity. From your perspective, to what extent do you think your campus has benefited from these new opportunities? How do you see the campus advancing the research mission and also advancing the teaching/learning mission?
4. **[Institutional change/transformation]** How does this initiative extend previous institutional goals and initiatives? Perhaps more importantly, how does this suggest the institution will work differently in the future? What kinds of institutional-wide changes or transformations are underway?
5. **[Sustainability]** Moving forward in the future (and the possibility of renewal of the grant), how do you think institutional name can sustain the campus initiatives that have been developed to produce more diverse biomedical researchers? What kinds of things will the campus put into place to ensure that this goal can be achieved when and if NIH funding shifts to other priorities?
6. **[Diversity commitment]** How do you see institutional commitment towards advancing diversity on this campus? Any challenges or strategies you have used to increase diversity in biomedical research fields? [Probe for institution, faculty, student levels].
7. Is there anything I did not ask about but you want to share about this campus and its role in expanding and diversifying the biomedical workforce? (e.g. Achievements, developments, vision).

**Semi-Structured Protocol – Deans or Associate Deans**

1. Please state your name and title for the recording. What is your role here at the institution relative to biomedical research, teaching and learning?
   1. Probes: Roles they serve can involve faculty development, undergraduate research programs, resources for facilities (new buildings, lab spaces), human resources (prioritizing teaching and learning with new hires), STEM-related endowments
   2. To what degree do you support faculty in your School/College in their efforts to improve degree completion and biomedical training? Can you share a specific example?
2. **[Impact]** NIH has made the decision to invest in institutions instead of only intervention programs. A large investment of xx million dollars was made in this campus to advance the progress and diversify the biomedical research workforce. From your perspective, what has been the impact on this campus of this investment? (Institution-wide changes, recognition, synergy with campus- planning for example).
3. How would you characterize the culture for innovation in teaching and learning in biomedical fields (social science if campus has initiative in that area) at this institution?
   1. Who would you identify are the key leaders in improving STEM teaching and learning at this institution? Why?
   2. How are [STEM] faculty incentivized to improve teaching and learning on this campus?
   3. How do you involve external stakeholders in the improvement of STEM teaching and learning?
   4. In what ways are STEM teaching and learning initiatives included as a priority among your fundraising and development goals?
4. **Advancing Student Success]** Given the diversity of students and the campus context, how has the campus been engaged in improving student graduation and movement into STEM careers? What remain key challenges?
5. **[Advancing Faculty Research]** This NIH initiative has a focus on improving faculty opportunities for research, advancing curricular change, and developing faculty capacity. From your perspective, what have been the benefits and how do you see the campus advancing the research mission and also advancing the teaching/learning mission?
6. **[Institutional change/transformation]** How does this initiative extend previous institutional goals and initiatives, and more importantly, how does this suggest the institution will work differently in the future? What kinds of institutional-wide changes or transformations are underway?
7. **[Sustainability]** Moving forward in the future (and the possibility of renewal of the grant), how do you think institutional name can sustain the campus initiatives that have been developed to produce more diverse biomedical researchers? What kinds of things will the campus put into place to ensure that this goal can be achieved when and if NIH funding shifts to other priorities?
8. Outside of external grant funding, what types of resource support do STEM programs receive?]
   1. Probes: Financial, academic, staffing, physical space, or other resources
   2. What is the process by which resources are allocated to STEM programs?
   3. To what extent are STEM programs funded by grants or institutional resources? What happens to programs when these grants end?
9. **[Diversity commitment]** How do you see institutional commitment towards advancing diversity on this campus? Any challenges or strategies you have used to increase diversity in biomedical or STEM research fields? [Probe for institution, faculty, student levels].
10. What are two of the more successful STEM support programs at your institution? How do you judge their success?
    1. Probes: Program goals, funding, type of students targeted, recruitment and admission procedures
    2. What were the identified needs that led to the development of these programs?
    3. From your vantage point, what are the mechanisms that allow STEM interventions to do well at your institution?
11. Besides the STEM support programs that you mentioned, what other resources does your institution offer for STEM students?
    1. Academic, financial, social, emotional, mentoring, professional development types of support
12. Is there anything I did not ask about but you want to share about this campus and its role in expanding and diversifying the biomedical workforce? (e.g. Achievements, developments, vision)
